# Synthesis, Characterization and Application of a MIP-polyHIPE for Selective Extraction of Angiotensin II Receptor Antagonists Residues in Natural Waters

**DOI:** 10.3390/ijerph20064878

**Published:** 2023-03-10

**Authors:** Andrea Speltini, Giancarla Alberti, Riccardo Rovida, Chiara Milanese, Giulia De Soricellis, Francesca Rinaldi, Gabriella Massolini, Angelo Gallo, Enrica Calleri

**Affiliations:** 1 Department of Chemistry, University of Pavia, via Taramelli 12, 27100 Pavia, Italy; 2 Department of Drug Sciences, University of Pavia, via Taramelli 12, 27100 Pavia, Italy; 3 Department of Chemistry, University of Turin, via Pietro Giuria 7, 10125 Turin, Italy

**Keywords:** polyHIPE, molecularly imprinted polymers, environmental waters, pharmaceuticals, pollutants, solid-phase extraction, water purification

## Abstract

Polymers via high internal phase emulsion (polyHIPEs) were molecularly imprinted with Irbesartan, an antihypertensive drug belonging to the class of angiotensin II receptor antagonists (sartan drugs), chosen for the proof-of-concept extraction of hazardous emerging contaminants from water. Different analyte-functional monomer molar ratios (1:100, 1:30 and 1:15) were investigated, and the MIP polyHIPEs have been characterized, parallel to the not imprinted polymer (NIP), by batch sorption experiments. The material with the highest template-functional monomer ratio was the best for Irbesartan removal, showing a sorption capacity fivefold higher than the NIP. Regarding the adsorption kinetics, the analyte–sorbent equilibrium was reached after about 3 h, and the film diffusion model best fitted the kinetic profile. Selectivity was further demonstrated by testing Losartan, another sartan drug, observing a fourfold lower sorption capacity, but still higher than that of NIP. The polymers were also synthesized in cartridges for solid-phase extraction (SPE), which was helpful for evaluating the breakthrough curves and performing pre-concentrations. These have been done in tap and river water samples (100–250 mL, 15–500 µg L^−1^ Irbesartan), obtaining quantitative sorption/desorption on the MIP-polyHIPE (RSD < 14%, *n* = 3). The NIP provided a recovery of just around 30%, evidence of partial uptake of the target from water.

## 1. Introduction

Molecularly imprinted polymers (MIPs) are synthetic materials capable of selective retention of a target molecule in preference to other closely related substances. MIP synthesis involves the co-polymerization of functional monomers and a cross-linker in the presence of a template molecule, i.e., the target analyte. The obtained materials are endowed with a highly cross-linked three-dimensional network reach in binding sites with shape, size and functionalities complementary to the target analyte and show good stability in a wide range of pH, solvents and temperatures; thus, they are claimed to be reliable substitutes for natural receptors and smart sorbent phases [1,2]. MIPs have found extensive application in analytical chemistry, including sensors [3,4,5] and chromatographic separations [6], and their use in numerous (complex) matrices for solid-phase extraction (SPE) is the most advanced technical application [1,2].

High internal phase emulsions (HIPEs) are a category of highly viscous emulsions in which an internal disperse phase makes up 74% of the whole volume [7]. These emulsions can be unstable and are subject to inversion and separation of the phases: these phenomena can be solved by using a suitable additive, one that promotes the formation of a convex interface between the two phases. HIPEs are created by mixing two liquids, one organic and the other aqueous: one is added to the other drop by drop, under continuous stirring. The stability of HIPEs is linked to several factors, including the molecular structure and polarity of the components, and the temperature. Adding a polymerization initiator to the external phase yields a highly porous polymer called polyHIPE [8]. PolyHIPEs were developed for several applications, such as chromatography, separation, sensing and drug delivery [8]. Although a number of applications have been exploited, limited progress has been made until now because of the poor mechanical stability of the material, mainly due to the extremely low foam density [9]. Consequently, adding porogenic solvents was considered to obtain materials with high surface area and better mechanical properties [10]. Acrylate-based polyHIPEs have been described and fully characterized [11,12].

Another weak point in applying polyHIPEs as sorbents is their low selectivity; this drawback can be solved by applying molecular imprinting technology, thereby developing MIP-polyHIPEs. This strategy has been adopted in the present work, aiming to prepare innovative adsorbents for the selective extraction of drugs that can be found as emerging contaminants in environmental waters. In particular, acrylic polyHIPEs [13] have been synthesized and molecularly imprinted with Irbesartan (IRB). This drug belongs to the class of sartans, an antihypertensive medication with a high production volume and structural similarities, including biphenyl, imidazole, or tetrazole groups. As a member of the class of pharmaceuticals and personal care products (PPCPs) and being partially removed in wastewater treatment plants [14], like other drugs, angiotensin II receptor antagonists or sartans are recognized emerging organic micropollutants, and their environmental diffusion is now under monitoring. However, little attention has been paid to these bioactive substances compared, for instance, to antimicrobials and anti-inflammatory drugs. As documented in recent research, sartans occurrence in environmental waters ranges from tens ng L^−1^ up to tens µg L^−1^ [14,15,16]. As newly highlighted by risk assessment studies, these concentration levels are responsible for non-negligible hazardous effects, with moderate–high environmental risk assigned to IRB [14].

To the Authors’ best knowledge, no report is currently available on the application of either polyHIPEs or MIP-polyHIPEs for extraction/removal/pre-concentration of IRB from water solution. Indeed, only one paper reports on using a MIP in conjunction with capillary zone electrophoresis to determine three sartans, including IRB, in human urine [17], and another one for Telmisartan in biological and formulation samples [18].

In this study, to take advantage of the MIPs’ selectivity combined with the peculiar structure of polyHIPEs, which shows great potential for in-flow adsorption processes, a molecularly imprinted polyHIPE was developed, characterized by several techniques and tested as a sorbent for IRB extraction from natural waters, as a proof-of-concept.

## 2. Materials and Methods

### 2.1. Chemicals and Materials

**Figure 1:**
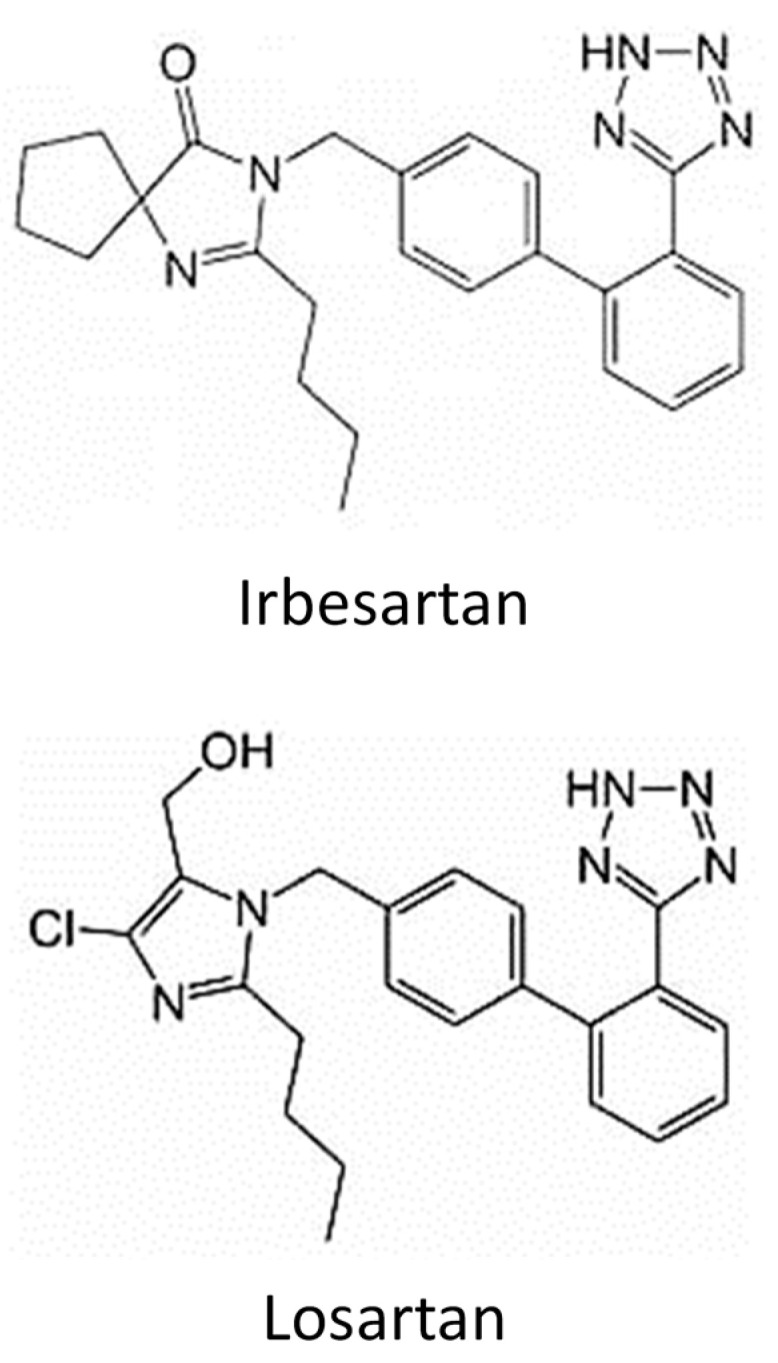
Molecular structures of Irbesartan (IRB) and Losartan (LOS).

Butyl acrylate, glycidyl methacrylate and trimethylolpropane triacrylate (TMPTA) were purchased from Sigma Aldrich (Milan, Italy). Synperonic PE/L121 was purchased from Croda Chemicals. Irgacure 819 was purchased from Ciba. All the reagents and analytical grade solvents, i.e., tetrahydrofuran (THF), methanol (MeOH) and acetonitrile (ACN), were used as received from the supplier (VWR, Milan, Italy). Double deionized water (DDW) was dispensed by a Q-POD purification system from Merck Millipore (Burlington, MA, USA). Degassed double-deionized water was achieved through nitrogen treatment. Empty polypropylene tubes (3, 6 mL capacity), polyethylene frits (20 µm pore size), trifluoroacetic acid (TFA, ≥99.0%), acetic acid (≥99.7%) and sodium acetate (≥99.0%) were purchased from Merck (Milan, Italy). HPLC gradient grade MeOH and ultrapure water were provided by VWR. IRB (>98.0%) TCI (Tokyo Chemical Industry) analytical standard was provided by Zentek S.r.l. (Milan, Italy). Pharmaceutical secondary standard-certified reference material Losartan (LOS) was obtained from Merck (Milan, Italy). IRB and LOS molecular structures are shown in Figure 1. The standard stock solutions of the two pharmaceuticals (1–1000 mg L^−1^) were prepared in MeOH and stored in the dark at 4 °C. Working solutions at lower concentrations were prepared daily by serial diluting of the concentrated solutions.

### 2.2. Synthesis of MIP-polyHIPE

The molecularly imprinted polyHIPEs were synthesized following a previously reported procedure but with photoactivated polymerization [19,20]. Briefly, the photoinitiator Irgacure 819 (140 mg, 0.33 mmol), butyl acrylate (4.3 mL, 30 mmol), the template IRB and the surfactant Synperonic PE/L121 (1.02 mL) were mixed under an inert atmosphere in a three-neck round bottom flask fitted with an overhead mechanical stirrer. The as-obtained oil phase was put under stirring (200 rpm) as glycidyl methacrylate (the functional monomer, 1.72 mL, 12.9 mmol) and trimethylolpropane triacrylate (0.96 mL, 3.6 mmol) were incorporated. Degassed double-ionized water (32 mL) was then poured into the dropping funnel and added dropwise to the mixture, while increasing the stirring speed to 300 rpm. Finally, when a cloudy solution was obtained, the mechanical stirring was set to 400 rpm until HIPE preparation was completed. Specifically, as water addition was achieved, the mixture was left stirring at room temperature under steady nitrogen flow for 1 h in the dark.

Finally, the creamy yellow emulsion was rapidly transferred into silicone rubber disk moulds and SPE cartridges to initiate the photoinduced polymerization directly. The latter was performed in the supports by irradiation using a 125 W UV lamp (HeliosItalquartz, Milan, Italy) covering a wavelength spectrum from 250 to 364 nm and peaking at 310 nm. Upon cooling to room temperature, the materials were subjected to preliminary washings to remove unreacted species, by-products and impurities; the polyHIPE disks were immersed for 20 min under constant stirring in double distilled water (2 × 50 mL), THF (2 × 50 mL) and MeOH (2 × 50 mL), sequentially, and the residual solvents were evaporated by air-drying under the hood until a constant weight was observed (48 h). The packed SPE cartridges were rinsed in flow, as described in Section 2.5.

### 2.3. Physicochemical Characterization of MIP- and NIP-polyHIPEs

Solid-state NMR ^1^H MAS and ^13^C CPMAS, SEM coupled with EDX, FT-IR, BET, DSC and TGA analyses were performed on the materials.

All solid-state NMR ^1^H MAS and ^13^C CPMAS spectra of MIP polyHIPE with IRB (pre washing), MIP-polyHIPE after template removal and IRB alone were recorded at room temperature on a JEOL ECZR 600 instrument, operating at 600.17 and 150.91 MHz for ^1^H and ^13^C nuclei, respectively. The samples were packed into cylindrical zirconia rotors with a 3.2 mm o.d. and a 60 µL volume. All ^1^H MAS experiments were acquired at 20 kHz after performing saturation recovery (for T_1_) measurements for relaxation optimization. ^13^C CPMAS spectra were acquired at a spinning speed of 20 kHz, using a ramp cross-polarization pulse sequence (90° ^1^H pulse of 2.2 µs; contact time of 3.5 ms for ^13^C) and an optimized recycle delay of 8 s (time domain in the direct dimension: 2048). For the ^13^C spectra, a two-pulse phase modulation (TPPM) decoupling scheme was used, with a radiofrequency field of 69.4 kHz.

Scanning electron microscopy (SEM) images were acquired by an EVO MA10 Scanning Electron Microscope, and the elemental analysis was performed by an Oxford XMax 50 mm^2^ detector coupled to the microscope. The measurements were conducted under an ultra-high vacuum with an electron generation voltage of 20 kV and a working distance of 8.5 mm.

Differential scanning calorimetry (DSC) was performed by a Q2000 apparatus (TA Instruments, New Castle, DE, USA) interfaced with a TA5000 data station by heating about 3 mg of powder in an open aluminium crucible from −20 °C to 350 °C and then cooling down to −20 °C (heating and cooling rate = 5 °C min^−1^) under nitrogen flow (50 mL min^−1^). Three independent measurements were acquired for each sample. The temperature accuracy of the instrument is ± 0.1 °C, the precision is ± 0.01 °C and the calorimetric reproducibility is ± 0.05%. DSC data were analysed using the Universal Analysis software by TA Instruments.

The thermogravimetric curves were acquired by a thermogravimetric analyser (TGA Q5000, TA Instruments Inc., USA) interfaced with a TA5000 data station by heating about 5 mg of sample in a Pt crucible under N_2_ flux (50 mL min^−1^) from 25 °C to 600 °C at 10 °C min^−1^. The data were analysed using the Universal Analysis software by TA Instruments, considering also the plot of the derivative of the weight with respect to temperature (DTG curve).

Infrared spectra were acquired at room temperature using a Nicolet FTIR iS10 spectrometer (Nicolet, Madison, WI, USA) equipped with Smart iTR with a diamond plate. Thirty-two scans in the 4000–600 cm^−1^ range at 4 cm^−1^ resolution were coadded.

The specific surface area of the samples was determined by a Sorptomatic 1990 equipment (ThermoElectron Corporation) operating with the static volumetric principle. About 150 mg of the sample was charged in the glass sample holder and degassed at 250 °C for 12 h. Subsequently, the samples were cooled down to −196 °C and an adsorption run was performed (B.E.T. method, analysing gas N_2_, 20 points for run, and blank done in He). The correction for the volume of the sample was performed by measuring He adsorption.

### 2.4. Analytical Characterization of MIP-polyHIPEs

The as-prepared materials (disk format) were cut into small pieces and, in the case of MIP-polyHIPE, first suspended in ca. 40 mL MeOH-TFA (99:1, *v*/*v*) to quantitatively remove the template (rotating plate, overnight).

Sorption isotherms were experimentally determined by a batch procedure, both on the washed MIP-polyHIPE and NIP-polyHIPE; about 15 mg of the polymer was placed in 10 mL PP tubes containing 5 mL acetate buffer solution (0.1 M; pH 5) at IRB initial concentrations ranging from 0 to 100 mg L^−1^ (*C*_0_) (rotating plate, overnight, and room temperature). The IRB sorbed amounts (*q*, mmol g^−1^) were calculated from the difference between *C*_0_ and the equilibrium concentration in the solution, *C*_eq_ (mol L^−1^).

The sorption kinetics were studied by contacting, for different time periods (rotating plate), 15 mg of polymer (after template removal) with a 15 mg L^−1^ IRB solution (5 mL, acetate buffer solution 0.1 M, pH 5) in 10 mL PP tubes.

The breakthrough curves were determined both on washed MIP-polyHIPE and NIP-polyHIPE in the packed-cartridge format; IRB solutions (25 mg L^−1^) were loaded on the cartridge (300 mg polymer) through a peristaltic pump (Miniplus3, Gilson, 0.5 mL min^−1^) and the fractions collected downstream the cartridge (10 mL each) were analysed for their drug content.

For this series of experiments, the concentration of IRB in aqueous solution was measured by UV–vis spectroscopy, using a Jasco V-750 spectrophotometer: 1 cm optical path quartz cuvette, λ_max_ 252 nm (ε 9800 M^−1^ cm^−1^), linearity range 0–20 mg L^−1^, limit of detection (LOD) 0.15 mg L^−1^ and limit of quantification (LOQ) 0.45 mg L^−1^.

### 2.5. Solid-Phase Extraction

The SPE tests were performed on MIP-polyHIPE (1:15) and NIP-polyHIPE materials in situ polymerized inside the PP cartridges. Each cartridge, 3 or 6 mL volume, was prepared by placing the pre-polymeric emulsion between two PE frits, thus obtaining a packed bed ~0.5 cm in height, corresponding to about 50 and 300 mg solid phase, respectively. Extraction was performed using a multi-position manifold (Resprep manifold, Restek Corporation, Bellefonte, PA, USA) connected to a vacuum pump. Before using the MIP-polyHIPE sorbent, the template was exhaustively removed by 40 mL MeOH-TFA (99:1, *v*/*v*), at a flow rate of ~2 mL min^−1^. The cartridges were first conditioned with 5 mL MeOH and 20 mL tap water; then, the sample (100/250 mL tap or river water fortified with known amounts of IRB) was loaded on the sorbent at a flow rate of around 2 mL min^−1^. After drying under vacuum for two minutes, elution was performed by 5 mL of MeOH-TFA (99:1, *v*/*v*) at a flow rate close to 0.5 mL min^−1^ to desorb the analyte efficiently. Before HPLC–UV analysis, the eluate collected in a 10 mL PP tube was evaporated to dryness under a N_2_ stream and reconstituted in 2 mL MeOH.

### 2.6. HPLC–UV Analysis

A new chromatographic method was developed to quantify IRB and LOS in the samples from SPE experiments. Chromatographic analysis was performed using an Agilent HPLC 1100 series system (Palo Alto, CA, United States) equipped with an XTerra MS C18 column (2.1 × 250 mm, 5 μm, 125 Å) from Waters (Milford, CT, United States), a quaternary pump, a Rheodyne injection valve (20 μL loop), a degasser, a UV–vis variable wavelength detector (λ = 220 nm) and thermostat oven (25 ± 0.5 °C).

Elution was carried out using (A) H_2_O and (B) ACN, both acidified with 0.1% formic acid (*v*/*v*), and setting isocratic conditions (65:35, *v*/*v*), with a flow rate of 0.3 mL min^−1^. In the reported conditions, IRB and LOS retention times were t_R_ = 7.9 min and t_R_ = 10.9 min, respectively.

The method was validated in terms of linearity through the construction of five-point calibration curves. Specifically, standard solutions in the range of 0.15–5 mg L^−1^ were prepared in MeOH by serial dilution of stock solutions (1 mg mL^−1^) of both pharmaceuticals in MeOH. A good linearity was obtained for both analytes: y = 307x − 33, R^2^ 0.9990 for IRB, and y = 673x − 4, R^2^ 0.9999 for LOS. LODs and LOQs were also assessed. For IRB, the LOD was 0.045 mg L^−1^ and LOQ 0.150 mg L^−1^; for LOS, the LOD was 0.027 mg L^−1^ and LOQ 0.089 mg L^−1^.

## 3. Results and Discussion

### 3.1. Synthesis and Physicochemical Characterization of the MIP- and NIP- polyHIPEs

MIP and NIP were prepared according to previously proposed procedures based on polyHIPE formation [19,20]. The monomers used were butyl acrylate, since its high glass transition temperature ensures good mechanical stability under solvent flow, trimethylolpropane triacrylate (cross-linker) to guarantee a high cross-linking density and consequent structural stability, and glycidyl methacrylate as a functional monomer. The latter has recently gained much attention in preparing stable spherical polymers [21,22,23]. Moreover, the possible opening of the glycidyl methacrylate’s epoxy ring could provide hydroxyl groups that can interact with template molecules [24].

Three different ratios of template (IRB):functional monomer (glycidyl methacrylate), were studied: 1:100, 1:30 and 1:15. The quantity of the functional monomer was progressively decreased, aiming to obtain a porous and selective polymer with a relatively high maximum sorption capacity comparable to that of classical acrylic-based molecularly imprinted polymers, and effectively applicable to separate and preconcentrate sartan drugs present in environmental waters at trace level.

**Figure 2:**
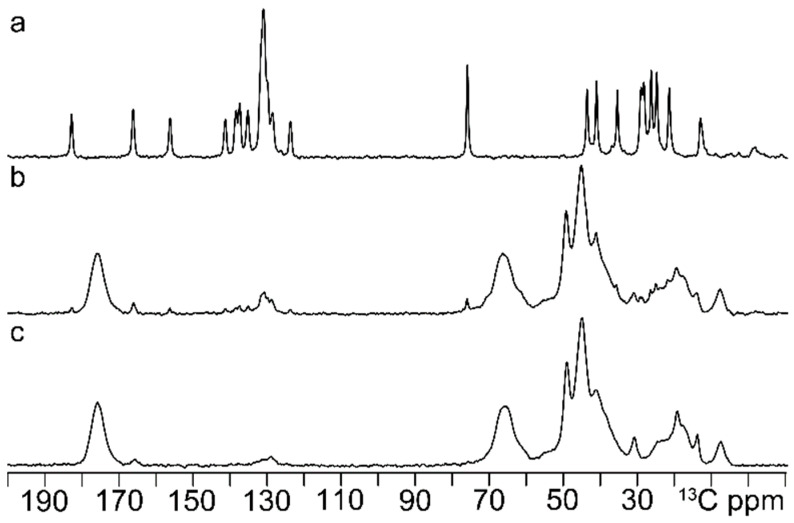
Stacking plot of ^13^C (150.91 MHz) CPMAS NMR spectra collected on (**a**) IRB, (**b**) polyHIPE with IRB (pre-washing) and (**c**) polyHIPE after template removal.

^13^C CPMAS experiments (Figure 2) were performed on the as-obtained MIP-polyHIPE, the same after washing (not containing IRB) and IRB alone. The spectrum acquired from the pure drug (Figure 2a) was compared to the MIP-polyHIPE spectra pre- (Figure 2b) and post-washing (Figure 2c). Irbesartan shows narrow lines (FWHM = 84 Hz) in the region typical of carbonylic and imminic carbons (above 150 ppm), aromatic carbon (120–140 ppm) and aliphatic carbon (from 10–70 ppm) compatible with the drug functional groups (see Figure 1). The dissolution of Irbesartan in the polymer induces a broadening of the sartan’s linewidths to 147 Hz (FWHM = 147 Hz), suggesting that the drug appears to be less crystalline and more amorphous, in agreement with its dispersion in the polymer. This is further confirmed by the small shift of several resonances and by the disappearance of the resonance at 13.4 ppm. Furthermore, this spectrum also reports on the polymer functional groups, suggesting the presence of the oxirane group in the polymer even after the synthesis. This is evinced by the peaks appearing at 66, 49, 46 and 42 ppm which correspond to those already observed for the oxirane group, and they do not correspond to those belonging to a diol group, in which case the chemical shifts would have been present only around 72 ppm [25].

By contrast, the spectrum of the template-free imprinted polyHIPE shows the disappearance of several narrow resonances at 182, 158, 78, 29 and 27 ppm, and it clearly suggests a highly efficient elimination of IRB. Therefore, the information obtained with ^13^C CPMAS experiments indicates that the washing procedure almost quantitatively eliminates the template (just a small residue probably remains in the polymer, as indicated by the peaks in the region at 130 ppm typical of compounds containing aromatic rings).

The infrared spectra of the same three samples and the one of NIP are reported in Figure S1 (Supplementary Material). The IRB spectrum (a) shows a complex signal between 3000 cm^−1^ and 2800 cm^−1^ due to the stretching of the C-H bonds (C-C-H and C=C-H), a band at 2361 cm^−1^ characteristic of the C-N bonds, a sharp peak at 1614 cm^−1^ for N-H bending, and a shoulder at 1565 cm^−1^ due to the aromatic C=C stretching and bending. The spectra for MIP (c) and NIP (d) are very similar. The spectrum recorded on the MIP sample before washing (b) shows the main character of the MIP patterns but in addition has an evident band due to the O-H bonds (blue line), a very well evident signal for the C-H stretching vibrations (red line), in particular at high wavenumbers, due to the benzenic hydrogen atoms, and the peak at 1614 cm^−1^ characteristic of the N-H bending. These last two features are characteristics of IRB and cannot be seen either in the MIP after template removal or in the NIP spectra. This findings prove that the washing step was almost quantitative and that no drug was covalently incorporated in the polymeric structure.

The calorimetric curves of pure IRB, MIP and NIP are reported in Figure S2 (Supplementary Material). As evident, in the IRB profile (a), a sharp endothermic peak attributable to melting (onset temperature = 182 °C) is followed by two exothermic signals due to decomposition (starting at 185 °C and 265 °C, respectively). The irreversibility of these last processes is testified by the absence of signals in the cooling curve. In the MIP and NIP samples, no traces of these signals can be seen. In particular, MIP (b) shows two small endothermic events centred at 118 °C and 244 °C, while NIP (c) is stable up to around 200 °C when a small endothermic peak (more energetic than in MIP) is evident.

The TGA curves of the three samples are reported in Figure S3 (Supplementary Material). IRB is thermally stable up to 185 °C; subsequently, it goes through three different mass loss steps (the second one starts at 265 °C and the last one at 340 °C), and at 500 °C it is almost fully decomposed (residual mass 5 wt%). The behaviour is fully in agreement with the DSC profile. The TGA curves for MIP and NIP samples are similar to each other, with a very small release of volatiles at temperatures lower than 120 °C and a subsequent huge mass release step starting from about 200 °C, that leads to the full decomposition of the samples at 600 °C. Differently from the IRB curve, for these samples, the TGA decomposition profile does not allow us to distinguish the evolution steps. Some differences can be highlighted between MIP and NIP by considering the mass loss values and the DTG curves: first of all, about 5 wt% of mass is released with a very small rate between 120 °C and 220 °C for the NIP sample, probably due to a slow release of volatiles in the pores of the sample. For the MIP sample, this step accounts only for 0.6 wt%. This behaviour can be linked to the higher surface area of NIP of 40 m^2^ g^−1^, namely 2.5 times the value for MIP (16 m^2^ g^−1^), which means a higher tendency to adsorb volatiles and gasses. As made evident by the DTG curve, the decomposition process is composed of three steps for the MIP sample, with the first one ending at 350 °C accounting for about 24.4% mass loss, the second one ending at 430 °C for 45.6% and the last one of 27.4%. The total mass release is 98% at 600 °C. On the contrary, for the NIP sample, the end of the first process is not distinguishable from the beginning of the second one, apart from a slight sloping change in the DTG, and they all account for a 79% mass loss (higher than the sum of the two steps for MIP). The third step accounts for a 16.5% mass loss. This means that the stability of the components is slightly different in the two structures.

**Figure 3:**
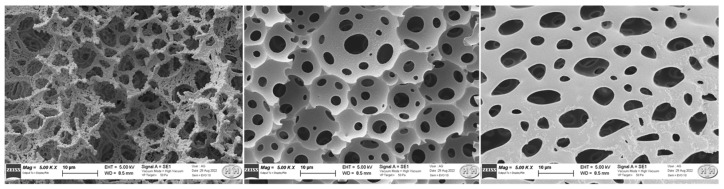
SEM images at different magnifications of the MIP-polyHIPE (after template removal).

SEM measurements were conducted on MIP polyHIPEs. Figure 3 shows the images of the MIP-polyHIPE after the template exclusion: the typical inner structure of polyHIPEs is maintained in the imprinted polymers. It has to be underlined that EDX (Tables S1 and S2) revealed C and O as the main components in both the MIP samples and in NIP, with very small amounts (≤ 0.15 atomic %) of P, Cl and Si. The technique was not able to detect N atoms in the MIP before template removal, due to the good dispersion of the drug in the polymeric matrix, also in accordance with the NMR findings, and hence the low concentration of these light atoms in the analysed spots (for the second row’s atoms, the detection by EDX is meaningful only for high amounts). Anyway, a higher C:O atomic ratio (2.5 vs. 2.3) is obtained in the as-prepared MIP-polyHIPE, suggesting the presence of a C-rich specie such as IRB together with the other HIPE precursors containing high amounts of O atoms.

### 3.2. Sorption Isotherms

Isotherm profiles were obtained for all prepared materials in acetate buffer solutions at pH 5 containing different quantities of the analyte IRB at 25 °C.

**Figure 4:**
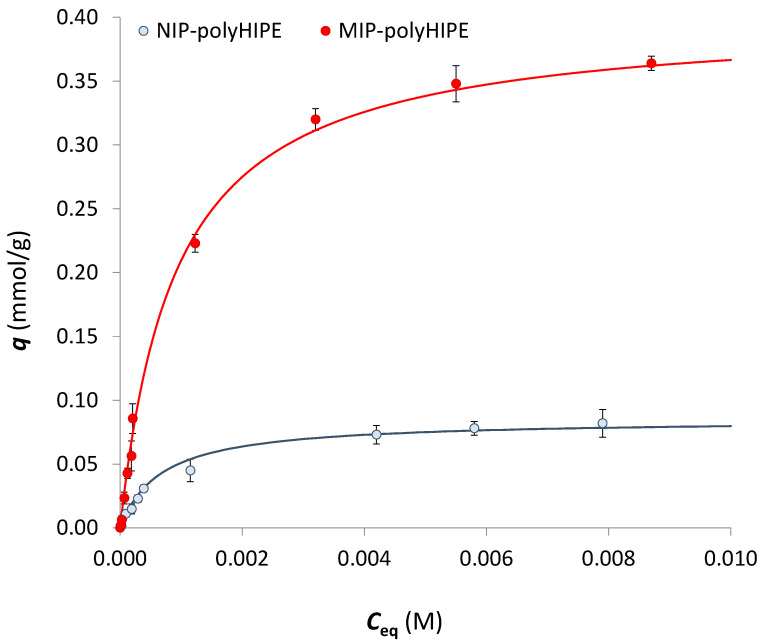
Sorption isotherm of IRB on the washed MIP-polyHIPE (1:15, red dots) and NIP-polyHIPE (grey dots). Discontinuous procedure: 15 mg of sorbent contacted with 0.1 M acetate buffer at pH 5 containing increasing amounts of IRB (from 8 µM to 8 mM). Continuous lines are the fitting by Langmuir equation. Experimental points are reported as average values of five replicates, and the error bars correspond to the standard deviation.

As an example, Figure 4 shows the sorption isotherms for the MIP-polyHIPE (1:15) and NIP-polyHIPE.

Well-known models of Langmuir and Freundlich were helpful in quantitatively describing the maximum uptake of the considered analyte under defined experimental conditions [26].

For all materials here analysed, the best fitting was obtained by applying the Langmuir model [27]:

(1) $$q=\frac{q_{\max}\cdot K_{L}\cdot C_{\mathrm{eq}}}{1+K_{L}\cdot C_{\mathrm{eq}}}$$

where *q* (mmol g^−1^) is the amount of the analyte at the equilibrium in the solid phase, *q*_max_ (mmol g^−1^) is the monolayer saturation sorption capacity, *K*_L_ (M^−1^) is the Langmuir constant and *C*_eq_ (M) is the analyte concentration at the equilibrium in the solution phase.

Table 1 summarizes the results obtained for the three MIP-polyHIPE materials and NIP-polyHIPE.

The MIP-polyHIPE polymer that demonstrates a better sorption capacity is the one with a template/functional monomer ratio of 1:15; this was predictable, given the higher concentrations of active sites compared to the other two. In terms of imprinting factor (IF), *viz*. the MIP-to-NIP sorption capacities ratio, the best-performing material shows an IF of 5, close to or even higher than the values usually considered suitable for MIPs applications [2].

The affinity constant remains almost unchanged between MIP and NIP since the analyte forms hydrogen bonds with the carboxyl groups of the functional monomer, whether pre-oriented as in the MIP-polyHIPEs or not as in NIP-polyHIPE.

It is important to highlight that the optimal ratio of functional monomer:IRB should probably be 1:5 or 1:3. Since this work aims to apply the MIP-polyHIPE to removing trace levels of sartans from water, a polymer with a sorption capacity of about 0.4/0.5 mmol/g, i.e., that prepared at a 15:1 ratio, has been considered sufficient for our purposes. Moreover, the sorption capacity (*q*_max_) obtained with the MIP-polyHIPE 1:15 is similar to the value previously obtained with a classical acrylic-based MIP for IRB (IRB: methacrylic acid: etilenglycoldimethacrylate = 1:4:20) [28,29]. The new material here described represents an advancement for its peculiar morphology, from an applicative point of view. In fact, the highly porous structure, which results in good solvent permeability, will allow its use in a flow system for drug removal in high-volume water samples.

### 3.3. Sorption Kinetics

**Figure 5:**
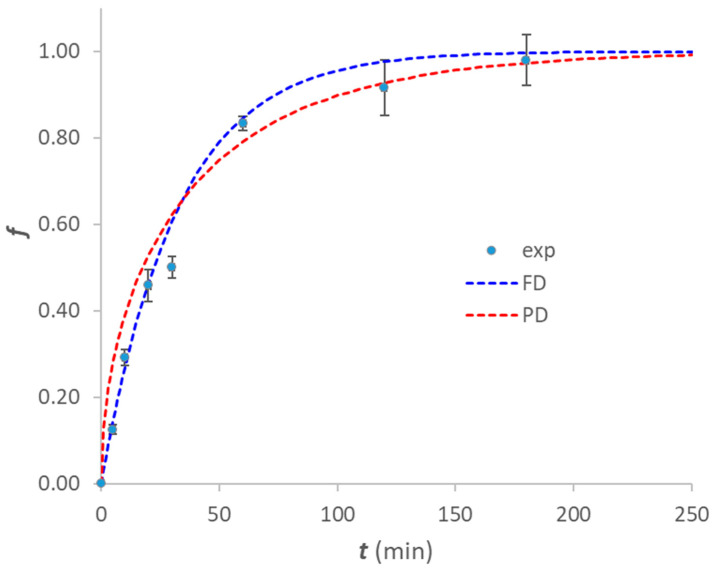
Sorption kinetics of IRB on MIP-polyHIPE (1:15) after template removal. Discontinuous procedure: 15 mg of sorbent contacted with 0.1 M acetate buffer at pH 5 containing IRB 12 µM for varying times. Dotted lines are the fitting by the HPDM model (homogeneous particle diffusion model): blue dotted line represents fitting by film diffusion, pseudo-first order equation; red dotted line represents fitting by particle diffusion, pseudo-second-order equation. Experimental points are reported as average values of five replicates, and the error bars correspond to the standard deviation.

The study of kinetic profiles helps evaluate the time needed to achieve equilibrium between the analyte and the solid phase in batch experiments. These tests were carried out by placing equal portions of MIP-polyHIPE in contact with 5 mL of 0.1 M acetate buffer solution at pH 5 containing IRB 12 µM for varying times. Figure 5 shows the kinetic profiles obtained for the MIP-polyHIPE (1:15) by plotting *f* (the fraction of analyte sorbed) vs. time (minutes). The HPDM model (homogeneous particle diffusion model) [19] was employed for the data fitting. Comparing the curves obtained by applying both the film diffusion, pseudo-first order equation and the particle diffusion, pseudo-second-order equation, it can be observed that the rate-limiting step is the analyte diffusion through the film surrounding the sorbent particles. Table 2 reports the kinetic constants and the correlation coefficients for both the applied fitting equations.

The time required to reach the equilibrium is about 4 h, i.e., it is necessary to wait at least 4 h to be sure the maximum concentration of analyte is sorbed (Figure 5).

### 3.4. Breakthrough Curves

Batch sorption experiments can be used to determine sorbents’ sorption capacity and study isotherms and kinetic sorption profiles. Nevertheless, in practical operation, continuous-flow fixed-bed columns are preferred; in these systems, the analyte concentration in the sorbent and solution phases varies in time and space. A quantitative approach is needed to design and optimize the fixed-bed column process, preferably matching all information derived from the batch experiments.

One of the main parameters to control during the development of a column method is the breakthrough volume, i.e., the volume at which a solute continuously introduced in a column begins to elute. In practice, this parameter is a function of the retention capacity of the sorbent and can be changed only by changing the solid phase.

The best-performing MIP-polyHIPE, i.e., prepared at the molar ratio 1:15, was selected as the sorbent in the packed-cartridge format for conventional SPE. The cartridges were easily prepared by in situ polymerization of the pre-polymeric mixture. Before use, the sorbent was placed on the vacuum manifold and washed in flow with acidic MeOH to remove the template (see Section 2.5).

**Figure 6:**
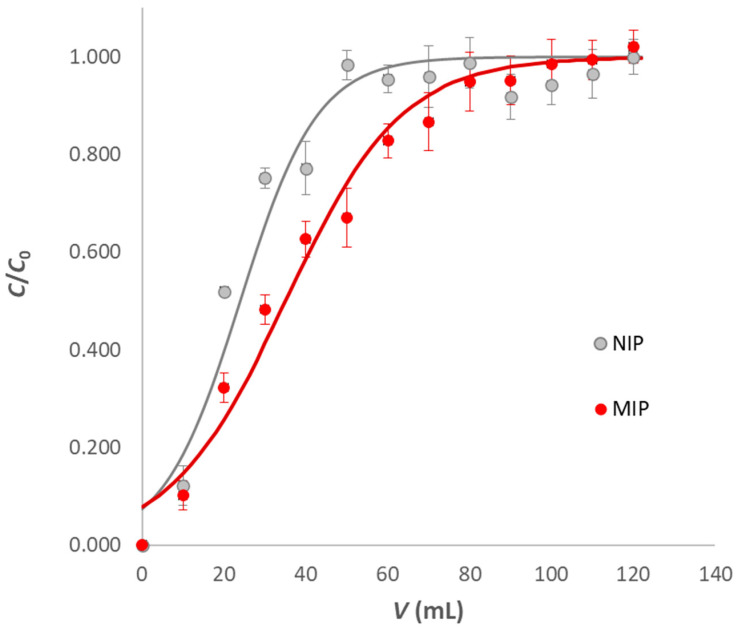
Breakthrough curves of IRB on the washed MIP-polyHIPE (1:15, red dots) and NIP-polyHIPE (grey dots). Experimental conditions: 120 mL 60 µM IRB solution (pH 5, 0.1 M acetate buffer), 6 cm^3^ SPE cartridge, 300 mg sorbent and flow rate 0.5 mL min^−1^. Continuous lines are the fitting by the Bohart–Adams model. Experimental points are reported as average values of five replicates, and the error bars correspond to the standard deviation.

The breakthrough volume was determined by loading 120 mL of 0.1 M acetate buffer at pH 5 solution containing 60 µM IRB on the SPE cartridge. An identical experiment was performed with a cartridge containing NIP-polyHIPE. The breakthrough curves obtained are shown in Figure 6.

In the case of MIP-polyHIPE, the analyte started overflowing after 100 mL, confirming what was previously found with sorption experiments. The breakthrough volume for the experiment with NIP-polyHIPE, corresponding to 60 mL, indicates that it did not perform quite as well as the MIP.

In order to predict breakthrough curves, the Bohart–Adams model was used. The model, originally developed by Bohart and Adams in a study on the absorption of chlorine by coal, is one of the most widely applied for a wide variety of cases. A simplified version is found in a fairly recent work by Chu [30]:

(2) $$\frac{C}{C_{0}}=\frac{1}{1+e^{\left[\frac{k_{\mathrm{BA}}}{\nu }\cdot \left(q_{0}\cdot w-C_{0}\cdot V\right)\right]}}$$

where *C* (M) is the analyte concentration at the eluted volume *V*, *C*_0_ (M) is the inlet concentration, *w* (g) is the amount of sorbent in the column, *k*_BA_ (M^−1^ min^−1^) is the Bohart–Adams constant, ν (mL min^−1^) is the flow rate and *q*_0_ (mmol g^−1^) is the maximum analyte concentration in the solid phase at the equilibrium, with the analyte concentration in solution equal to *C*_0_.

The Bohart–Adams constants obtained for MIP-polyHIPE and NIP-polyHIPE are, respectively, 1391(256) M^−1^ min^−1^ and 1405(406) M^−1^ min^−1^. Analogous to what was observed for the isotherms, the constants’ similarity can be attributed to the hydrogen bonds of the IRB with the carboxyl groups of the functional monomer, whether pre-oriented as in the cavities of the MIP-polyHIPE or not as in NIP-polyHIPE.

### 3.5. Analytical Application for Solid-Phase Extraction

The first SPE experiments were carried out on 6-mL capacity cartridges containing ca. 300 mg of MIP-polyHIPE (1:15), prepared as described above. With the aim of investigating the potentiality of this new material to selectively sequester IRB present in real environmental waters, the SPE tests were straightway focused on tap water samples (250 mL) spiked with 500 µg L^−1^ IRB. Tap water, collected from the Pavia municipal waterworks and representative of natural waters, was chosen because of its constant composition, pH and greater similarity to surface water than ultrapure water.

The results showed a complete sorption of the contaminant from water at the native pH and a subsequent quantitative release in the eluting solution, with recovery higher than 90% (see Table 3) and an enrichment factor of 50. The same trials performed on the NIP-polyHIPE yielded a partial recovery, around 30%, due to the lower sorption capacity than that of the imprinted material, as verified by analysis of the percolated solution collected downstream of the cartridge. This outcome further attests to the successful imprinting, in addition to the results collected from sorption isotherms and breakthrough curves (see previous sections).

Another crucial test was performed under the same conditions on a tap water sample spiked with 500 µg L^−1^ IRB in the presence of LOS, a structural analogue of IRB belonging to the sartan family (Figure 1), to evaluate a possible competition between the two compounds for the MIP’s cavities. It was observed that LOS was partially retained on the sorbent (around 30%), and this indicates that (1) the MIP-polyHIPE is not specific but selective for molecules of similar structures; (2) the sorbent material shows an affinity for the sartan congeners, suggesting a possible application for multianalyte extraction from water, under optimized conditions.

These findings were further corroborated by the data relative to the sorption capacity determined for Losartan, i.e., 0.12 mmol g^−1^ on the MIP-polyHIPE, which resulted in ca. threefold lower value than that for IRB (see Section 3.2. and Table 1) but fourfold higher compared to the value found on the NIP-based solid phase.

Another series of experiments were undertaken on smaller cartridges containing about 50 mg polymer to operate in the micro-SPE mode [31]. These tests were performed at lower IRB concentrations both on tap water (spikes 15 and 100 µg L^−1^) and not tampered river water (spike 15 µg L^−1^) collected from the Ticino River near Pavia (Italy).

As reported in Table 3, recovery was quantitative, underlining a complete removal of IRB from water—with unchanged extraction efficiency in going from tap to raw surface water—and a complete elution from the cartridge.

The overall enrichment factor for the above-reported extractions was up to 125, and good inter-day precision was achieved for all experiments, with residual standard deviation (RSD) < 14% (*n* = 3).

Based on these results, it is reasonable to assume that coupling the pre-concentration step to more sensitive instrumental techniques such as LC–MS would also allow suitable sensitivity for routine monitoring analyses at the ng L^−1^ levels. Moreover, the findings here collected open the way for the future development of an analytical method that, after full validation, could be applied for sartans determination in water.

## 4. Conclusions

The results from this pilot study highlight that the new MIP polyHIPE shows good selectivity for the target molecule over closely related compounds, with sorption capacity significantly higher compared to that of the not imprinted material, and suitable for the quantitative adsorption of IRB from water, from the low µg L^−1^ to the mg L^−1^ levels. As a remarkable advantage, the MIP-polyHIPE reveals itself as a double-purpose material for future in-batch or in-flow applications, i.e., the removal of IRB (and generally sartans) from contaminated natural waters and analytical pre-concentration, in the packed-cartridge micro-SPE format, for trace determination of IRB.

## Figures and Tables

**Table 1:** Parameters of the Langmuir isotherms for MIP-polyHIPEs at different ratios of IRB:functional monomer (1:100; 1:30; and 1:15) and NIP-polyHIPE (average values obtained from five isotherms for each material. Numbers in parentheses are the standard deviation on the last digit). Experimental conditions: 15 mg sorbent in 5 mL of 0.1 M acetate buffer at pH 5 containing increasing amounts of IRB (from 8 µM to 8 mM). T = 25 °C.

Sorbent	*q*_max_ (mmol/g)	*K*_L_ (M^−1^)
MIP-polyHIPE (1:100)	0.05(2)	2.5(4)∙10^3^
MIP-polyHIPE (1:30)	0.23(7)	2.1(6)∙10^3^
MIP-polyHIPE (1:15)	0.42(5)	2.1(4)∙10^3^
NIP-polyHIPE	0.08(4)	2.5(6)∙10^3^

**Table 2:** Sorption kinetics of IRB on MIP-polyHIPE (1:15). Experimental conditions: 15 mg of sorbent contacted with 0.1 M acetate buffer at pH 5 containing IRB 12 µM for varying times. R = correlation coefficient. Numbers in parentheses are the standard deviation on the last digits. T = 25 °C.

Fitting Model	*k* (min^−1^)	R
Film diffusion $f=1-e^{-k\cdot t}$	0.031(2)	0.996
Particle diffusion $f=\sqrt{1-e^{-2\cdot k\cdot t}}$	0.008(1)	0.983

**Table 3:** Mean recovery of IRB from natural waters by MIP-polyHIPE SPE.

Sample	Sorbent Amount (mg)	Spike (µg L^−1^)	Mean Recovery (%) ^a^	
			MIP-polyHIPE	NIP-polyHIPE
Tap water ^1^ (250 mL)	300	500	94(13)	30(4)
Tap water ^1^ (100 mL)	50	100	95(12)	-
Tap water ^1^ (100 mL)	50	15	96(11)	-
River water ^2^ (100 mL)	50	15	95(12)	-

^1^ pH 7.7, conductivity at 20 °C 270 µS cm^−1^, 5 mg L^−1^ Cl^−^, 0.6 mg L^−1^ NO_3_^−^, 5 mg L^−1^ SO_4_^2−^, 36.1 mg L^−1^ Ca^2+^, 8.1 mg L^−1^ Mg^2+^ and 12.5 mg L^−1^ Na^+^. ^2^ pH 7.4, conductivity at 20 °C 178 µS cm^−1^, 5.3 mg L^−1^ Cl^−^, 3.8 mg L^−1^ NO_3_^−^, 23.9 mg L^−1^ SO_4_^2−^, 86 mg L^−1^ HCO_3_^−^, 27.3 mg L^−1^ Ca^2+^, 6 mg L^−1^ Mg^2+^, 5.1 mg L^−1^ Na^+^, 1.4 mg L^−1^ K^+^ and SiO_2_ 3.6 mg L^−1^. ^a^ the standard deviation is reported in parentheses (*n* = 3).

## Data Availability

Not applicable.

## Supplementary Materials

The following supporting information can be downloaded at: https://www.mdpi.com/article/10.3390/ijerph20064878/s1, Figure S1: IR spectra recorded on IRB (a), as-obtained MIP (b), MIP after template removal (c) and NIP (d).; Figure S2: Calorimetric profiles for IRB (a), MIP after template removal (b) and NIP (c); Figure S3: TGA and DTG profiles for IRB (a), MIP after template removal (b) and NIP (c); Table S1: EDX results for the MIP sample before template removal; Table S2: EDX results for the MIP sample after template removal.

## References

[1] Martín-Esteban A. (2016). Recent molecularly imprinted polymer-based sample preparation techniques in environmental analysis. Trends Environ. Anal. Chem..

[2] Speltini A., Scalabrini A., Maraschi F., Sturini M., Profumo A. (2017). Newest applications of molecularly imprinted polymers for extraction of contaminants from environmental and food matrices: A review. Anal. Chim. Acta.

[3] Ahmad O.S., Bedwell T.S., Esen C., Garcia-Cruz A., Piletsky S.A. (2019). Molecularly imprinted polymers in electrochemical and optical sensors. Trends Biotechnol..

[4] Leibl N., Haupt K., Gonzato C., Duma L. (2021). Molecularly imprinted polymers for chemical sensing: A tutorial review. Chemosensors.

[5] Alberti G., Zanoni C., Losi V., Magnaghi L.R., Biesuz R. (2021). Current Trends in Polymer Based Sensors. Chemosensors.

[6] Song Z., Li J., Lu W., Li B., Yang G., Bi Y., Arabi M., Wang X., Ma J., Chen L. (2022). Molecularly imprinted polymers based materials and their applications in chromatographic and electro-phoretic separations. Trends Anal. Chem..

[7] Lissant K.J. (1966). The geometry of high-internal-phase-ratio emulsions. J. Colloid Interface Sci..

[8] Silverstein M.S. (2014). PolyHIPEs: Recent advances in emulsion-templated porous polymers. Prog. Polym. Sci..

[9] Menner A., Powell R., Bismarck A. (2006). Open porous polymer foams via inverse emulsion polymerization: Should the definition of high internal phase (ratio) emulsions be extended?. Macromolecules.

[10] Cameron N.R. (2005). High internal phase emulsion templating as a route to well-defined porous polymers. Polymer.

[11] Tripodo G., Marrubini G., Corti M., Brusotti G., Milanese C., Sorrenti M., Catenacci L., Massolini G., Calleri E. (2018). Acrylate-based poly-high internal phase emulsions for effective enzyme immobilization and activity retention: From computationally-assisted synthesis to pharmaceutical applications. Polym. Chem..

[12] Corti M., Calleri E., Perteghella S., Ferrara A., Tamma R., Milanese C., Mandracchia D., Brusotti G., Torre M.L., Ribatti D. (2019). Polyacrylate/polyacrylate-PEG biomaterials obtained by high internal phase emulsions (HIPEs) with tailorable drug release and effective mechanical and biological properties. Mater. Sci. Eng. C.

[13] Speltini A., Tripodo G., Rinaldi F., Massolini G., Profumo A., Calleri E. (2022). Carbon nanotubes-modified poly-high internal phase emulsions for pharmaceuticals pre-concentration and determination. J. Pharm. Biomed. Anal..

[14] Lopez F.J., Pitarch E., Botero-Coy A.M., Fabregat-Safont D., Ibáñez M., Marin J.M., Peruga A., Ontañón N., Martínez-Morcillo S., Olalla A. (2022). Removal efficiency for emerging contaminants in a WWTP from Madrid (Spain) after secondary and tertiary treatment and environmental impact on the Manzanares River. Sci. Total Environ..

[15] Ślósarczyk K., Jakóbczyk-Karpierz S., Różkowski J., Witkowski A.J. (2021). Occurrence of pharmaceuticals and personal care products in the water environment of Poland: A Review. Water.

[16] Oberleitner D., Schmid R., Schulz W., Bergmann A., Achten C. (2021). Feature-based molecular networking for identification of organic micropollutants including metabolites by non-target analysis applied to riverbank filtration. Anal. Bioanal. Chem..

[17] Zhang M., Wei F., Zhang Y.F., Nie J., Feng Y.Q. (2006). Novel polymer monolith microextraction using a poly (methacrylic acid-ethylene glycol dimethacrylate) monolith and its application to simultaneous analysis of several angiotensin II receptor antagonists in human urine by capillary zone electrophoresis. J. Chromatogr. A.

[18] Mudiam M.K.R., Chauhan A., Singh A.K., Sharma V.P., Saxena P.N. (2016). Molecularly imprinted SPE combined with dispersive liquid–liquid microextraction for selective analysis of telmisartan in biological and formulation samples. Bioanalysis.

[19] Brusotti G., Calleri E., Milanese C., Catenacci L., Marrubini G., Sorrenti M., Girella A., Massolini G., Tripodo G. (2016). Rational design of functionalized polyacrylate-based high internal phase emulsion materials for analytical and biomedical uses. Polym. Chem..

[20] Kimmins S.D., Wyman P., Cameron N.R. (2012). Photopolymerised methacrylate-based emulsion-templated porous polymers. React. Funct. Polym..

[21] Ganewatta N., El Rassi Z. (2018). Monolithic capillary columns consisting of poly (glycidyl methacrylate-co-ethylene glycol dimethacrylate) and their diol derivatives with incorporated hydroxyl functionalized multiwalled carbon nanotubes for reversed-phase capillary electrochromatography. Analyst.

[22] Feczkó T., Trif L., Németh B., Horák D. (2016). Silica-coated poly (glycidyl methacrylate-ethylene dimethacrylate) beads containing organic phase change materials. Thermochim. Acta.

[23] Jiang J., Zhang Y., Guo X., Zhang H. (2012). Ambient temperature synthesis of narrow or monodisperse, highly cross-linked, and “living” polymer microspheres by atom transfer radical precipitation polymerization. RSC Adv..

[24] Wan Y., Wang M., Fu Q., Wang L., Wang D., Zhang K., Xia Z., Gao D. (2018). Novel dual functional monomers based molecularly imprinted polymers for selective extraction of myricetin from herbal medicines. J. Chromatogr. B.

[25] Sankar S.S., Lonikar S.V., Gilbert R.D., Fornes R.E., Stejskal E.O. (1990). Solid-state *CPMAS* ^13^C-NMR studies of the reaction of an epoxy resin with masked isocyanates. J. Polym. Sci. B Polym. Phys..

[26] Alberti G., Amendola V., Pesavento M., Biesuz R. (2012). Beyond the synthesis of novel solid phases: Review on modelling of sorption phenomena. Coord. Chem. Rev..

[27] Langmuir I. (1916). The constitution and fundamental properties of solids and liquids. Part I. Solids. J. Am. Chem. Soc..

[28] Zanoni C., Rovida R., Magnaghi L.R., Biesuz R., Alberti G. (2022). Voltammetric Detection of Irbesartan by Molecularly Imprinted Polymer (MIP)-Modified Screen-Printed Electrodes. Chemosensors.

[29] Rovida R., Zanoni C., Alberti G., Magnaghi L.R., Biesuz R. MIP-based screen-printed electrode for Irbesartan sensing. Proceedings of the 3rd International Electronic Conference on Applied Sciences (ASEC 2022).

[30] Chu K.H. (2010). Fixed bed sorption: Setting the record straight on the Bohart-Adams and Thomas models. J. Hazard. Mater..

[31] Ghorbani M., Aghamohammadhassan M., Chamsaz M., Akhlaghi H., Pedramrad T. (2019). Dispersive solid phase microextraction. Trends Anal. Chem..

