# Supplementary material for: Synthesis, Characterization and Application of a MIP-polyHIPE for Selective Extraction of Angiotensin II Receptor Antagonists Residues in Natural Waters
Source: Int J Environ Res Public Health. 2023 Mar 10;20(6):4878. doi: 10.3390/ijerph20064878 (PMC10049482; doi:10.3390/ijerph20064878)
Supplement: Supplementary file 1 [file ijerph-20-04878-s001.zip › ijerph-2211539-supplementary.pdf]

Supplementary Material

Synthesis, characterization and application of a MIP-polyHIPE for selective extraction of angiotensin II receptor antagonists residues in natural waters

Andrea Speltini, Giancarla Alberti, Riccardo Rovida, Chiara Milanese, Giulia De Soricellis, Francesca Rinaldi, Gabriella Massolini, Angelo Gallo, and Enrica Calleri

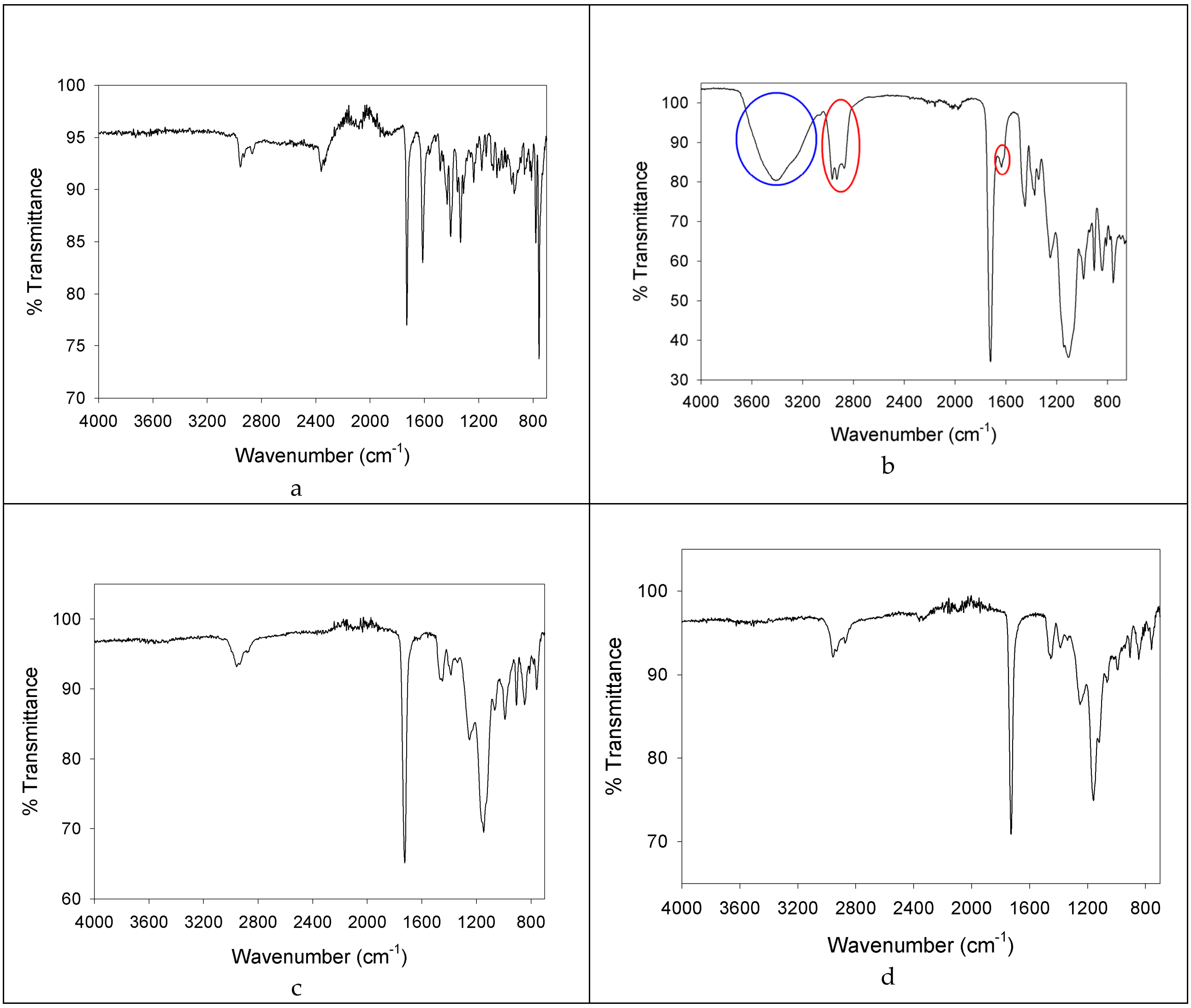

Figure S1. IR spectra recorded on IRB (a), as-obtained MIP (b), MIP after template removal (c), and NIP (d).

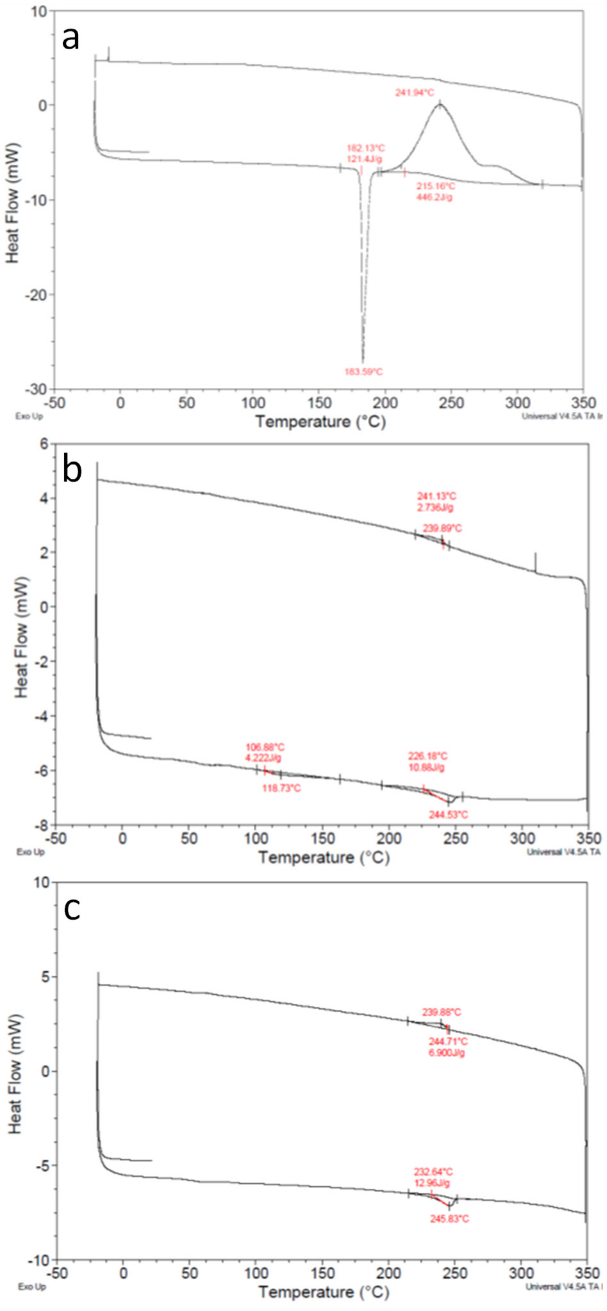

Figure S2. Calorimetric profiles for IRB (a), MIP after template removal (b) and NIP (c).

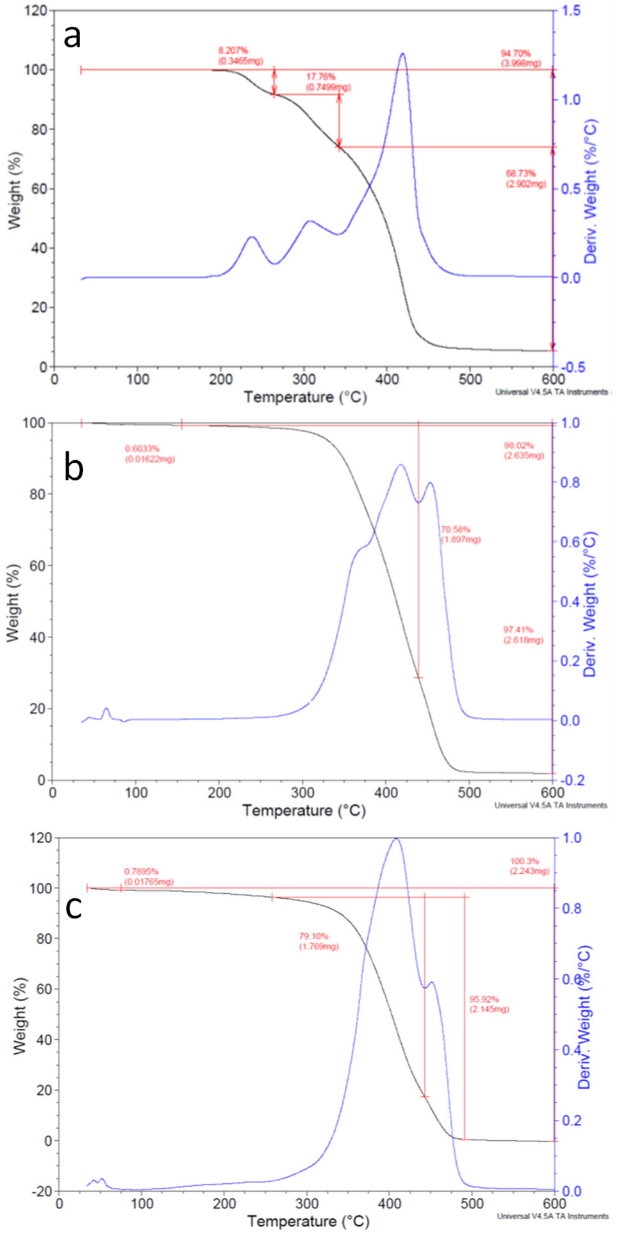

Figure S3. TGA and DTG profiles for IRB (a), MIP after template removal (b) and NIP (c).

Table S1. EDX results for the MIP sample before template removal: example of two characteristic points (the pictures of the analysed zones are on the right of the table – the powders for EDX analyses are not sputtered with gold).

Element	App Conc.	Intensity Corrn.	Weight%	Weight% Sigma	Atomic%
C K	60.72	1.3055	64.80	0.68	71.21
O K	10.90	0.4387	34.59	0.69	28.54
P K	0.33	1.3289	0.35	0.06	0.15
Cl K	0.15	0.8268	0.26	0.06	0.10

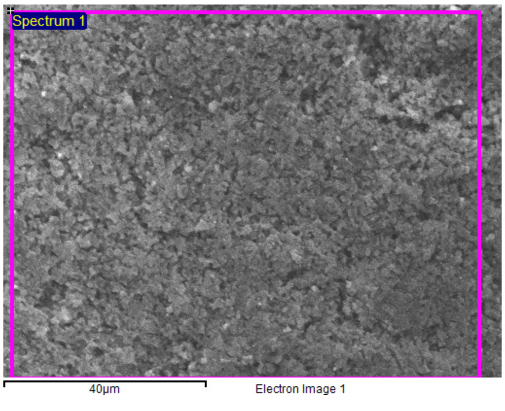

Element	App Conc.	Intensity Corrn.	Weight%	Weight% Sigma	Atomic%
Totals			100.00		

Element	App Conc.	Intensity Corrn.	Weight%	Weight% Sigma	Atomic%
C K	85.32	1.3623	64.69	0.49	71.01
O K	14.99	0.4418	35.03	0.49	28.87
P K	0.36	1.3270	0.28	0.04	0.12
Totals			100.00		

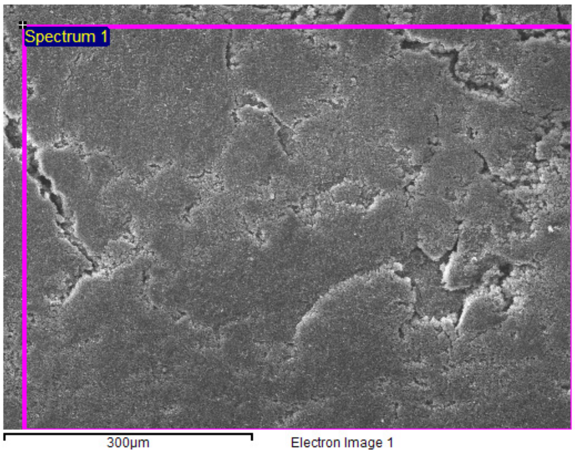

Table S2. EDX results for the MIP sample after template removal: example of two characteristic points (the pictures of the analysed zones are on the right of the table).

Element	App Conc.	Intensity Corrn.	Weight %	Weight % Sigma	Atomic %
C K	104.33	1.3406	63.82	0.50	70.25
O K	19.54	0.4479	35.78	0.50	29.57
Si K	0.27	0.9247	0.24	0.03	0.11
P K	0.25	1.3216	0.15	0.03	0.07
Totals			100.00		

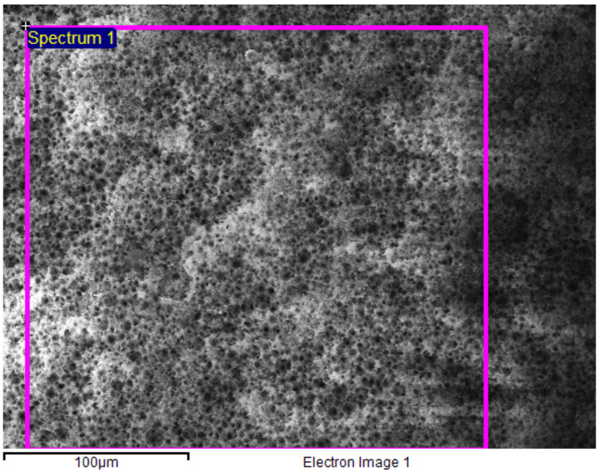

Element	App Conc.	Intensity Corrn.	Weight %	Weight % Sigma	Atomic %
C K	105.10	1.3589	63.94	0.44	70.32
O K	19.39	0.4477	35.81	0.44	29.56
Si K	0.16	0.9243	0.14	0.03	0.07
P K	0.18	1.3229	0.11	0.03	0.05
Totals			100.00		
